# 435. Evaluation of Fecal Pharmacokinetics and Metagenomic Changes Following the Administration of Intravenous Omadacycline to Healthy Subjects

**DOI:** 10.1093/ofid/ofae631.149

**Published:** 2025-01-29

**Authors:** Jinhee Jo, Chenlin Hu, Travis J Carlson, John C Williamson, Yolanda T Belin, Anne J Gonzales-Luna, Kevin W Garey

**Affiliations:** University of Houston, Houston, Texas; University of Houston, Houston, Texas; The University of Texas at Austin College of Pharmacy, San Antonio, Texas; Atrium Health Wake Forest Baptist, Winston-Salem, NC; Atrium Health Wake Forest Baptist, Winston-Salem, NC; Department of Pharmacy Practice and Translational Research, University of Houston College of Pharmacy, Houston, TX; University of Houston College of Pharmacy, Houston, TX

## Abstract

**Background:**

Omadacycline (OMC), an aminomethylcycline antibiotic, has potent *in vitro* activity against *C. difficile* (CD) and has been associated with a low propensity to cause CD infection when used to treat other infections. A recent phase 1 study of oral OMC demonstrated its protective gut microbiome profile, despite achieving high fecal concentrations, compared to oral vancomycin in healthy subjects. This study aimed to investigate the fecal pharmacokinetics and gut microbiome changes of intravenous (IV) OMC following its administration to healthy volunteers.

**Methods:**

Healthy adults aged 18 to 40 years consented to receive a 10-day course of OMC: 200 mg of IV on day 1, followed by 100 mg once daily on days 2-5, then 300 mg orally once daily on days 6-10. Daily stool samples were collected and metagenomic analysis targeting 16S ribosomal RNA gene was performed using the Miseq platform (Illumina). Fecal concentrations of OMC were measured using liquid chromatography, mass-spectrometry assay.

**Results:**

Eight healthy volunteers aged 30±4 years (male: 50%; body mass index: 27.1±4.3 kg/m^2^) were enrolled. By day 2, 87.5% (7/8) of stool samples had detectable OMC concentrations >40 mcg/g stool, increasing to >100 mcg/g stool by day 5. The maximum OMC concentrations were observed on day 10 (mean 853.5 mcg/g stool). Metagenomic analysis revealed preservation of the Bacteroidota phylum (+0.5±0.3%; p=0.06) and a non-significant increase of the Proteobacteria phylum (+0.9±0.6%; p=0.1). Significant increases were observed in the Actinobacteriota and Verrucomicrobiota phyla (+1.1±0.3%; p< 0.05 and +1.7±0.5%; p< 0.0001, respectively). These changes were driven by the *Bifidobacteriaceae* and *Akkermansiaceae* families, respectively. A significant decrease in the Bacillota phylum (-5.1±0.7%; p< 0.0001) was observed, primarily in the *Lachnospiraceae*, *Ruminococcaceae*, and *Streptococcaceae* families.

**Conclusion:**

Intravenous OMC achieved high fecal concentrations that exceeds the minimum inhibitory concentration 90 of CD while preserving key bacterial species in the gut. Studies investigating changes in bile acids and short-chain fatty acids corresponding with these metagenomic changes are warranted.

**Disclosures:**

**Travis J. Carlson, PharmD, BCIDP**, Aimmune Therapeutics, Inc.: Speakers Bureau **John C. Williamson, PharmD**, Armata Pharmaceuticals: Grant/Research Support|Blue Collar Vaccines and Therapeutics: Board Member|Blue Collar Vaccines and Therapeutics: Ownership Interest|Paratek Pharmaceuticals: Grant/Research Support|ST Pharm Co, Ltd: Grant/Research Support **Anne J. Gonzales-Luna, PharmD, BCIDP**, Ferring Pharmaceuticals: Advisor/Consultant|Innoviva Specialty Therapeutics: Advisor/Consultant|Merck and Co: Grant/Research Support|Paratek Pharmaceuticals: Grant/Research Support|Seres Therapeutics: Grant/Research Support **Kevin W. Garey, MS;PharmD**, Paratek Pharmaceuticals: Grant/Research Support

